# Effect of intermittent theta-burst stimulation on the thyroid and adrenal systems in resistant depressed patients

**DOI:** 10.1192/j.eurpsy.2024.289

**Published:** 2024-08-27

**Authors:** F. Duval, V. Danila, T. Weiss, F. Gonzalez Lopera, M. Tomsa

**Affiliations:** ^1^Psychiatry, Centre Hospitalier APF2R, Rouffach, France

## Abstract

**Introduction:**

Disturbances in the hypothalamic-pituitary-thyroid (HPT) and hypothalamic-pituitary-adrenal (HPA) axes have been frequently reported in treatment resistant depressed patients (TRDs). So far, the effects of intermittent theta-burst stimulation (iTBS) treatment—a form of repetitive transcranial magnetic stimulation (rTMS) technique—on the activity of the HPT and HPA axes are poorly understood.

**Objectives:**

The present study aimed to evaluate the effects of iTBS sessions, applied to the left dorsolateral prefrontal cortex, in TRDs with abnormal chronobiological HPT functioning at baseline (BL) possibly associated with hypercortisolemia.

**Methods:**

The ∆∆TSH test (i.e., the difference between the thyrotropin response to protirelin tests [∆TSH] performed at 8 AM and 11 PM on the same day) and the dexamethasone suppression test (DST) were performed in 12 TRDs and 14 healthy hospitalized control subjects (HCs). To be enrolled in this study, patients had to show at BL reduced ∆∆TSH values (i.e., < 2.5 mU/L) and a score of 18 or greater on the 17-item Hamilton Rating Scale for Depression (HAMD-17). Post-DST cortisol maximum (COR_max_) serum level in excess of 120 nmol/L defined DST non-suppression (i.e., hypercortisolemia)—6 TRDs were DST non-suppressors at BL. After 10 and 20 iTBS sessions the ∆∆TSH test and the DST were repeated in all inpatients. A positive clinical response was defined by a final HAMD-17 score ≤ 8.

**Results:**

Compared to HCs, ∆∆TSH values were lower in TRDs at BL (p < 0.00001), and remained reduced after 10 and 20 iTBS sessions (p < 0.001 and p < 0.02 respectively). Post-DST COR_max_ levels were higher in TRDs than in HCs at BL (p < 0.01), but were comparable to those of HCs after 10 and 20 iTBS sessions. Responders (n = 5) were characterized by 1) a normalization of their ∆∆TSH values after 20 iTBS sessions (whereas after 10 iTBS sessions ∆∆TSH values were still reduced compared to HCs [p < 0.05]), and 2) a normality of post-DST COR_max_ levels at BL—while after 10 and 20 iTBS sessions post-DST COR_max_ levels were decreased compared to HCs (p < 0.006 and p < 0.03 respectively). Non-responders (n = 7) showed 1) no significant change in their ∆∆TSH values which remained lower than those of HCs at each assessment (all p < 0.001), 2) while increased post-DST COR_max_ levels found at BL (p < 0.0008 vs. HCs) normalized from the 10th iTBS session.

**Conclusions:**

The present pilot study suggests that successful iTBS treatment can restore the chronobiological activity of the HPT axis. Although iTBS may increase glucocorticoid receptor signaling, baseline hypercortisolemia could negatively impact subsequent response to iTBS treatment.

**Disclosure of Interest:**

None Declared

